# Dynamic Testing of Lime-Tree (*Tilia Europoea*) and Pine (*Pinaceae*) for Wood Model Identification

**DOI:** 10.3390/ma13225261

**Published:** 2020-11-20

**Authors:** Anatoly Bragov, Leonid Igumnov, Francesco dell’Isola, Alexander Konstantinov, Andrey Lomunov, Tatiana Iuzhina

**Affiliations:** 1 Research Institute for Mechanics, National Research Lobachevsky State University of Nizhny Novgorod, 603950 Nizhny Novgorod, Russia; bragov@mech.unn.ru (A.B.); fdellisola@gmail.com (F.d.); constantinov.al@yandex.ru (A.K.); lomunov@mech.unn.ru (A.L.); yuzhina_tatiana@mech.unn.ru (T.I.); 2 Research and Education Mathematical Center “Mathematics for Future Technologies”, 603950 Nizhny Novgorod, Russia

**Keywords:** timber, natural composite, Kolsky method, deformation diagrams, wood species, energy absorption, wood model, verification

## Abstract

The paper presents the results of dynamic testing of two wood species: lime-tree (*Tilia europoea*) and pine (*Pinaceae*). The dynamic compressive tests were carried out using the traditional Kolsky method in compression tests. The Kolsky method was modified for testing the specimen in a rigid limiting holder. In the first case, stress–strain diagrams for uniaxial stress state were obtained, while in the second, for uniaxial deformation. To create the load a gas gun was used. According to the results of the experiments, dynamic stress–strain diagrams were obtained. The limiting strength and deformation characteristics were determined. The fracture energy of lime and pine depending on the type of test was also obtained. The strain rates and stress growth rates were determined. The influence of the cutting angle of the specimens relative to the grain was noted. Based on the results obtained, the necessary parameters of the wood model were determined and their adequacy was assessed by using a special verification experiment.

## 1. Introduction

Wood is a complex natural composite. It is widely used as a material for damping intensive dynamic loads of shock or explosive nature. In order to conduct reliable numerical analysis of the designs of containers for transporting hazardous substances, using wood as shock damping components, reliable mathematical models are required that take into account its complex multicomponent structure of wood. The actively developing models of wood deformation and destruction can be used in various design complexes to simulate the behavior of technically complex structures that incorporate wood elements. However, the complex nature of wood means that not a single model can be used for all purposes, thus different models must be used to solve various specific problems. In order to reliably describe the behavior of wood in a dynamically loaded structure, its model should include a sufficiently large set of parameters that take into account the deformation anisotropy and the dependence of the strength properties on the strain rate, density, temperature and moisture content. For example, in the manual for the LS-DYNA software package, the wood model No. 143 has 29 parameters: five modules for transversely isotropic constitutive equations, six tensile strengths for yield criteria, four hardening parameters to peak stresses, eight softening parameters after peak, and six parameters speed effect [1]. The LS-DYNA calculation complex library contains model parameters for only two wood species: southern yellow pine and Douglas fir. This necessitates detailed studies of various wood species for various types of stress–strain states in a wide range of strain rates and temperatures, in order to equip mathematical models of wood with parameters (identification) that can adequately describe the behavior of the engineering structure containing wood components under shock wave loads.

The first detailed review of using wood as an element of damping structures was given by Johnson [2]. In the report, he noted that the dynamic properties of wood are not well understood. In the past three decades, quite a lot of works have appeared that explored various aspects of the high-speed deformation and destruction of wood, including the use of wood as a damping material in containers for transportation of radioactive materials by air, road and rail transport [3]. A large amount of research into the dynamic properties of wood was performed by Reid et al. [4,5,6]. In these works, the dependences of destructive stress and energy absorption on the impact velocity were obtained.

It was noted that dynamic destructive stresses are several times higher than static ones. The authors also postulated that failure stresses of specimens fabricated along the grain are an order of magnitude higher than that of the specimens cut transverse to the grain. The relationship between mechanical parameters of selected wood species (*Carya* sp., *Fagus sylvatica* L., *Acer platanoides* L., *Fraxinus excelsior* L., *Ulmus minor* Mill.) and the grain orientation angle concern loading direction was investigated in [7]. It was observed that as the angle between fibers and loading direction increases whereas the mechanical characteristics of all wood species decrease.

In the works of Bragov et al., using the Kolsky method, dynamic diagrams of birch, aspen, and sequoia were obtained at strain rates of ~10^3^ s^−1^ with different direction of cutting specimens relative to the direction of timber fibers [8,9]. The deformation diagrams of specimens under loading along the grain are much higher than those under loading transverse to the grain. The limiting deformative characteristics have the opposite direction.

In recent years, interest in studying the effect of moisture, density and cutting angle on the mechanical properties of wood has increased significantly. Adalian et al. [10] and Eisenacher et al. [11] carried out a cycle of tests on wood in the range of strain rate from 10^−3^ to 10^3^ s^−1^. Wouts et al. [12], Ha et al. [13,14] and Hao et al. [15] considered the damping ability of various biological materials of plant origin from the point of view of improving artificial damping structures. Mach et al. [16] calculated the hysteresis losses (dissipated energy) as the area bounded by the loading and unloading curves, whereas stored energy (recoverable energy) is defined as the area under the unloading curve. Thus, the total energy is calculated by summing the area of the hysteresis loss and the stored energy. In the work of Novikov et al. [17], a large number of tests of sequoia, birch, aspen, and pine was carried out at different cutting angles, temperatures ranged from −30 °C to +65 °C, and at the moisture content of 5%, 20% and 30%. As a result, the dependence of strength on moisture and cutting angle was determined.

The mechanical properties of wood strongly depend on the place of growth, its age, the place of cutting and the type of stress–strain state. Therefore, the results obtained by different authors may differ significantly from each other. The purpose of this work is to conduct a detailed study of the effect of strain rate and type of stress–strain state on the mechanical properties of wood as well as to identify and verify the wood model.

## 2. Test Methods, Materials and Specimens

**Figure 1:**
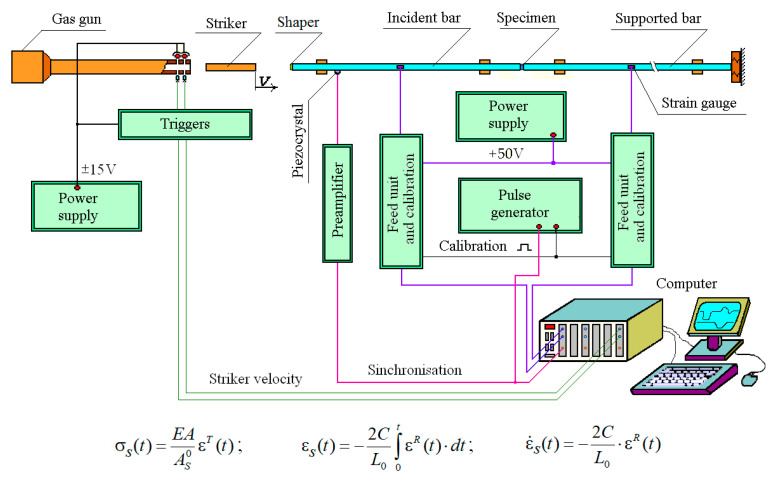
Installation scheme and basic dependencies.

For dynamic compressive tests of wood, the Kolsky method and split Hopkinson pressure bar (SHPB) technology were used [18]. Figure 1 shows the installation diagram, as well as the main formulas for determining the parametric dependencies of deformation, stress and strain rate of the sample under compression. Two measuring bars were made of high-strength aluminum alloy and had a diameter of 20 mm and length of 1.5 m. During testing, a striker accelerated in the barrel of a gas gun impacts the SHPB and excites an elastic compression wave in the loading measuring bar, which, upon reaching the specimen, deforms it. The second (support) measuring bar acts as a dynamometer-waveguide and allows you to register the transmitted strain pulse ε*^T^*(*t*) and then to determine the process of stress developing in the specimen. The pulse ε*^R^*(*t*) reflected from the specimen in the loading bar makes it possible to determine the process of strain rate change in the specimen, and its integration allows to determine the process of the specimen deformation development. Small-length foil strain gages were used for registration elastic strain pulses in the bars. Based on these pulses, the parametric processes of stress, strain and strain rate over time are calculated using the formulae shown in the lower part of Figure 1. Then, excluding time as a parameter, a dynamic stress–strain curve is constructed with the known law of variation of the strain rate [19].

Dynamic tests were carried out with specimens of lime-tree (*Tilia europoea*) and pine (*Pinaceae*) with a diameter of 20 mm and a length of 10 mm, cut from solid wood at an angle of 0° and 90° relative to the axis of the tree trunk. The flat ends of the samples were carefully hand-grinded before testing. The physical parameters of tested materials are shown in the Table 1.

The total amount of tested samples of each wood species amounted 50 pieces: 40 pieces were used for mechanical testing and 10 pieces were used for subsequent wood model verification. In each test mode during mechanical testing (strain rate and loading direction), 4–5 experiments were carried out, the results of which were then averaged. All the experiments were carried out in laboratory conditions at room temperature and 50% air humidity.

## 3. Experimental Results

**Figure 2:**
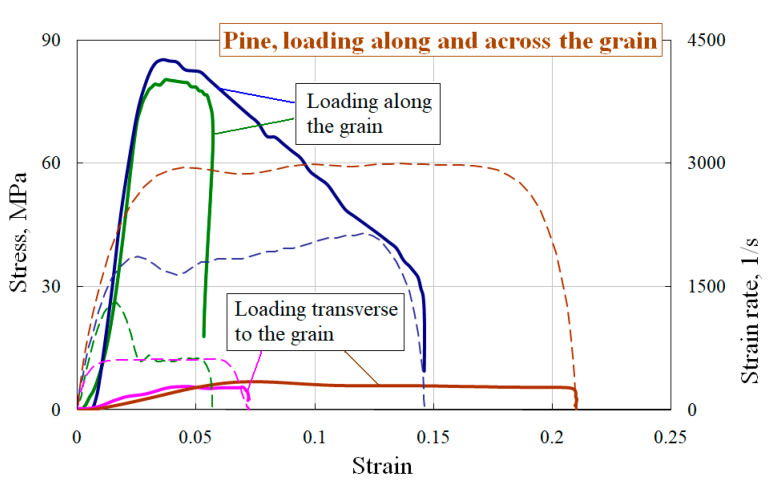
Deformation diagrams of pine under loading along and across the grain.

**Figure 3:**
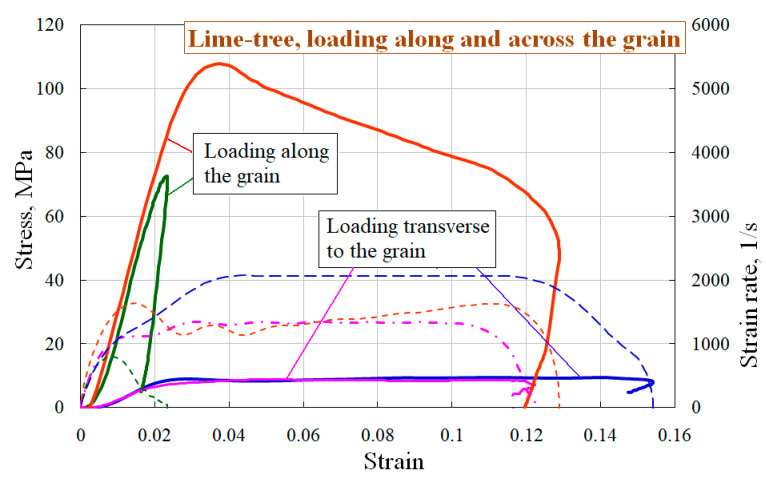
Deformation diagrams of lime-tree under loading along and across the grain.

Using the above methods, the dynamic tests of pine and lime-tree were carried out. As a result, their dynamic stress–strain curves, the ultimate strength and deformative characteristics of the specimens cut along and transverse to the grain were obtained. Dynamic strain diagrams for pine and lime-tree under uniaxial stress state are presented in Figure 2 and Figure 3. The figures show the averaged stress–strain curves and appropriate strain rate-strain curves. The spread of the obtained properties was no more than 6%. In all figures solid lines show the dependences of the true stress of the specimen on its deformation σ~ε, and the dotted lines (in the lower part of the diagrams) correspond to the history of the strain rate change έ~ε (the appropriate axis is on the right).

Two test modes on strain rate were chosen: with non-destructive and destructive results. From the obtained stress–strain diagrams, it follows that for both wood species the specimens cut and loaded along the grain have the largest modulus of the load branch in the diagram, as well as ultimate stress and energy absorption.

**Figure 4:**
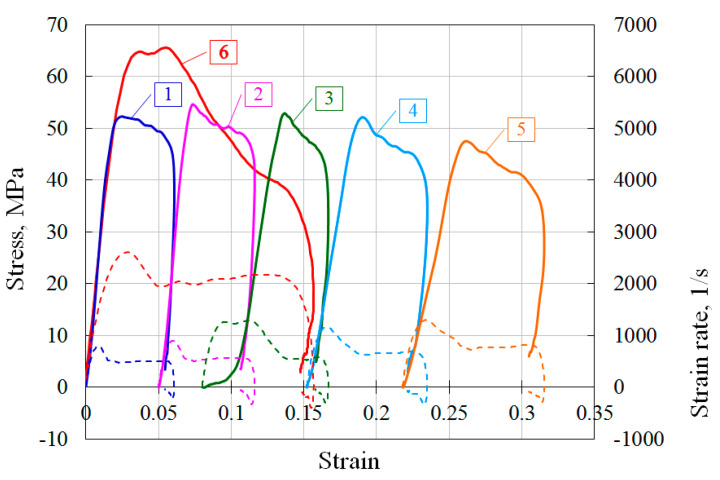
An example of multiply repeated loading of the specimen with its minor damage.

Wood endurance can be indicated in Figure 4, where diagrams of fivefold loading of a pine specimen with its minor damages (curves 1–5) and a single independent loading of another similar specimen with its complete failure (curve 6) are presented. The diagrams are located on the deformation axis conditionally in order to make it easier to assess the effect of multiple loading on the steepness of the load sections of the stress–strain curves. The average strain rate at repeated loads was ~600–800 s^−1^, and in the case of specimen failure, the strain rate was about 2200 s^−1^. One can clearly see a decrease in the steepness of the load section of stress–strain curve during repeated loads by a factor of 2–3, which is associated with partial destruction of the specimen during each loading and violation of the flatness of its ends.

**Figure 5:**
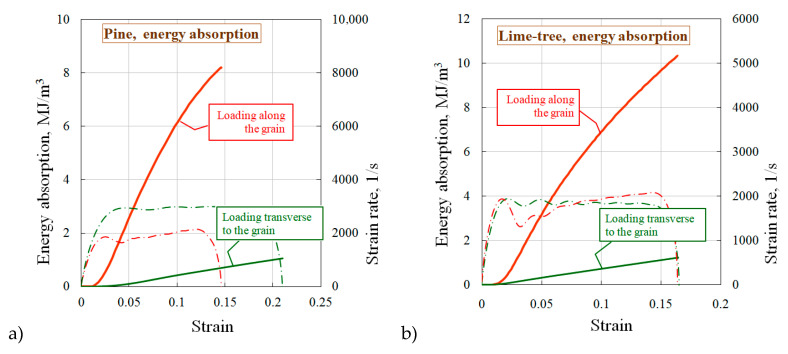
Energy absorption of the tested wood species: (**a**) for pine, (**b**) for lime-tree.

As an important characteristic of the timber damping capacity, the energy absorption of the tested wood species was estimated under loading along and transverse to the grain by calculating the area under the curve σ~ε (Figure 5). 

For both wood species, there is a significant excess of energy absorption by the specimens cut and tested along the grain, compared to the specimens cut and tested transverse to the grain.

It is interesting to compare the results on the damping ability of wood-based materials obtained by other researchers. In the work [12] the damping capacity of two types of deciduous and coniferous wood: beech and spruce under loading in the longitudinal, tangential and radial directions were compared. The highest specific energy absorbed was noted for specimens under longitudinal loading, and the smallest—under tangential loading. This is traditional tendency for wood materials. 

While in [14], the damping ability of the mesocarp layer in durian shell (tropical fruit) was investigated and, as a result of the research, it was found the inverse effect: specific energy absorption of the mesocarp layer under lateral loading is higher than that under axial one. This may be due to the fact that the durian shell does not have the same clearly pronounced fiber structure as in wood, therefore the authors’ accentuation on the lower strength and damping ability of the material in the axial direction of load application compared to lateral loading refers to the radial and tangential strength of shell of this fruit.

## 4. Identification and Verification of the Wood Model

To describe the behavior of wood under dynamic loads in the library of the LS-DYNA calculation complex, there is model No. 143 MAT_WOOD. This is a model of a transversely isotropic material for solid elements. It is possible to set material properties or use a predefined set of constants, but only for two species of pine growing in the USA: southern yellow pine and Douglas fir. 

The primary features of the model are:

- Transverse isotropy for the elastic constitutive equations (different properties are modeled parallel and perpendicular to the grain).
- Yielding with associated plastic flow formulated with separate yield (failure) surfaces for the parallel- and perpendicular-to-the-grain modes.
- Hardening in compression formulated with translating yield surfaces.
- Post-peak softening formulated with separate damage models for the parallel and perpendicular-to-the-grain modes.
- Strength enhancement at high strain rates.

Model 143 for pine contains a predefined set of 29 parameters depending on moisture content and temperature. According to the results of the pine tests, some parameters of this model were adjusted for a particular batch of samples under study. These parameters are shown in Table 2.

It should be noted that the elastic moduli of pine under loading along and across the grain were obtained as a result of quasi-static tests, since the Kolsky method, in principle, does not allow constructing a stress–strain curve in which the slope dσ/dε of the elastic loading section would be equal to the static modulus of elasticity. Usually the steepness of this section is several times less than the static Young’s modulus. The reasons for this are as follows:

- The difference in the cross-sectional areas of the measuring bars and the sample (some part of the incident wave is reflected from the free surface around the sample), causing an apparent deformation of the sample,
- The presence of gaps between the ends of the measuring bars and the sample (due to the non-parallelism of its ends and their roughness or insufficient “pressing” of the sample during the preparation of the test),
- Dispersion during the propagation of waves in the bars (decrease in the steepness of the transmitted pulse during the propagation time to the recording strain gauge).

In order to confirm the adequacy of the model parameters determined from experiments, it is necessary to verify it, but in experiments other than those in which these parameters were obtained. For this purpose, we used a model experiment in full-scale and numerical implementation. The simulation of the indentation process was performed using the finite element method. The explicit time integration scheme was used for solving the equations of motion in time. Modeling was performed using the Dynamics-2^®^ software package [20]. The numerical experiment was simulated in an axisymmetric formulation, and its design corresponded to a similar natural test.

**Figure 6:**
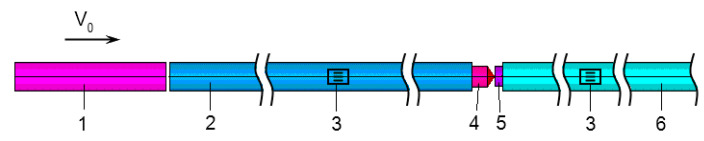
High speed indentation experiment scheme.

To verify the model of material behavior, we used an experimental scheme for dynamic indentation by using the system of a split Hopkinson bar. The indicated procedure is schematically shown in Figure 6. The sample 5 and a removable indenter 4 with a hemispherical head are sandwiched between the measuring bars 2 and 6. Upon impact loading by the striker 1, a compression impulse is generated in the bar 2 the duration of which depends on the length of the striker and the amplitude on the speed of the striker. In the case of hemispherical indenter, the contact area at the initial moment of indentation is very small, therefore, the amplitude of the reflected pulse is very significant, and so the sample is loaded several times. For reliable recording of additional loading cycles, it is necessary to increase the length of the supporting bar in comparison with the length of the loading bar as much as required to register loading cycles [21].

**Figure 7:**
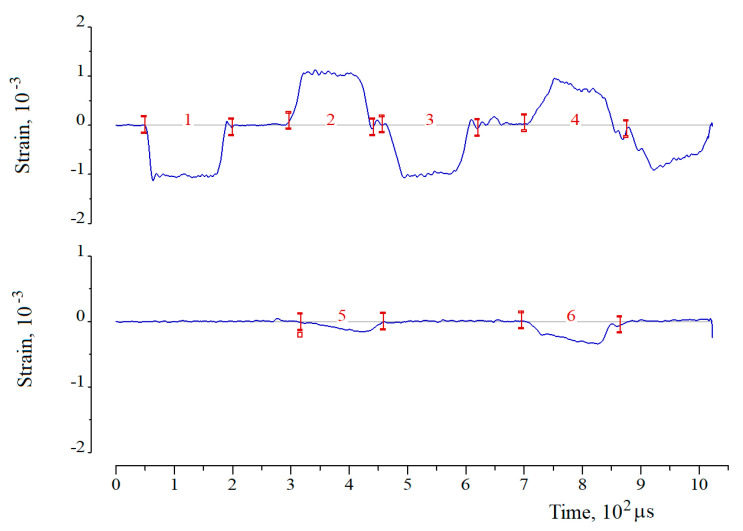
Typical waveform obtained in the experiment for high-speed indentation, taking into account additional cycles: **upper beam**—data from bar 2; **lower beam**—data from bar 6.

Using strain gauges 3, elastic strain pulses are recorded in the measuring bars (Figure 7). The following pulses are indicated by numbers: 1—incident (loading) strain pulse ε*^I^*(*t*), 2—reflected pulse in the first loading cycle $\varepsilon_{1}^{R}(t)$, 3—incident (loading) strain pulse $\varepsilon_{2}^{I}(t)$ in the second loading cycle, 4—reflected strain pulse in the second loading cycle $\varepsilon_{2}^{R}(t)$, 5—transmitted pulse (first cycle) $\varepsilon_{1}^{T}(t)$, 6—transmitted pulse (second cycle) $\varepsilon_{2}^{T}(t)$.

**Figure 8:**
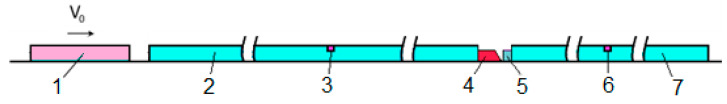
High speed indentation experiment simulation scheme.

The loading of the SHPB system in the numerical experiment (Figure 8) is performed in the same way as in the natural experiment—with the help of a striker having an initial velocity *V*_0_. In the calculation process, the incident and reflected strain pulses are calculated in element 2 and the past—in element 6. The time dependences obtained during the simulation are compared with the corresponding values recorded during the experiment.

The indenter is made of high-strength tungsten–cobalt alloy and is modeled by a rigid non-deformable material.

To assess the processes occurring in the sample, data obtained from measuring bars is used. In accordance with the Kolsky formulas, one can calculate the law of change of the indenter penetration rate into the sample *V*(*t*) and the penetration resistance force *F*(*t*):

*V*(*t*) = *C*_1_·(ε*^I^*(*t*) + ε*^R^*(*t*)) − *C*_2_·ε*^T^*(*t*) *F(t)* = *E_2_*∙*S_2_* ε*^T^*(*t*). (1)

here ε*^I^*(*t*), ε*^R^*(*t*) and ε*^T^*(*t*) denote the incident, reflected, and transmitted strain pulses in the measuring bars, respectively, *E* is the elastic modulus, and *C* is the bar velocity of sound. The subscripts 1 and 2 refer to the first (loading) and second (supporting) measuring bars.

**Figure 9:**
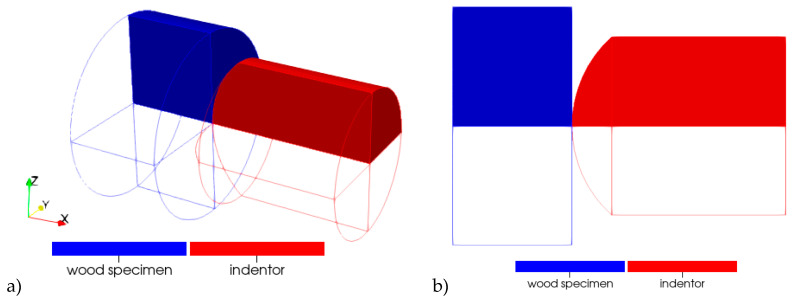
Geometric statement of the problem of numerical simulation: (**a**) 3D configuration, (**b**) plane configuration.

When modeling the process of dynamic indentation into wood samples, the following scheme was used: a sample and an indenter were considered (Figure 9). The spatial discretization of the indenter and the sample was done by using a solid three-dimensional finite element with one integration point. Due to the presence of symmetry, a quarter of the geometric model was considered. On the planes of symmetry, the corresponding boundary conditions were specified: at the nodes lying on the plane with the normal in the direction of the oY axis, zero velocities in the oY direction and zero velocities of rotation about the oX and oZ axes were set; at the nodes lying on the plane with the normal in the direction of the oZ axis, zero velocities in the oZ direction and zero velocities of rotation about the oX and oY axes were set.

Zero velocities in the direction of the oX axis were set at the nodes belonging to the surface of the sample, which in full-scale experiments rested against the transmitted measuring bar.

The indenter was modeled by an absolutely rigid undeformable body. For the indenter, the law of the change in the speed of its movement in the axial direction was set. The time dependence of indenter’s axial velocity for a particular experiment was calculated using the Equation (1) based on the signals recorded in the measuring bars.

The “surface–surface” contact interaction was set between the indenter and the sample.

In natural experiments on the indentation the samples of pine were used in the form of tablets with a length of 10 mm and a diameter of 20 mm. Some of the samples were cut in the direction of the wood grain, while another part of the samples was cut in the perpendicular to the grain direction.

**Figure 10:**
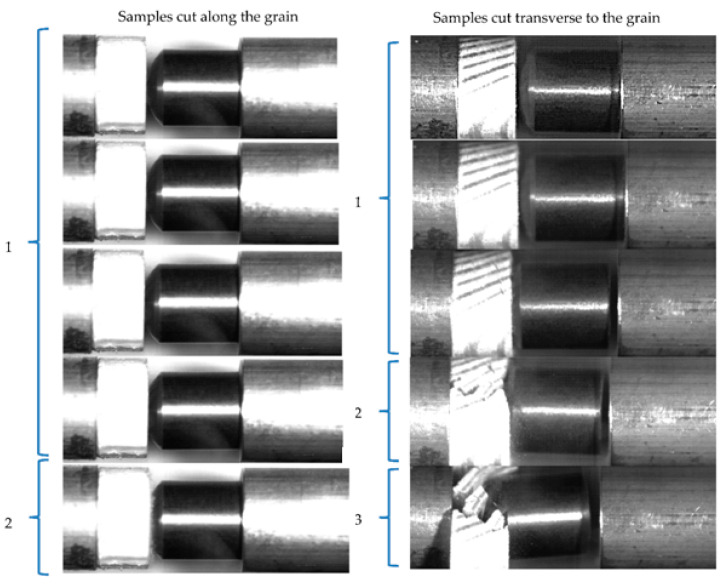
High-speed registration of dynamic indentation.

As mentioned above, in the process of dynamic indentation, the sample is subjected to multiple loading in the experiment (see Figure 7), thus undergoing a certain deformation in each loading cycle. High-speed film recording of the indentation process makes it possible to estimate a number of loading cycle and indentation depth at which the destruction of the material occurs. Figure 10 shows the frames of such registration, made using a high-speed camera HSFC Pro.

The left part of the figure corresponds to the experiment with the sample cut in the direction along the grain, whereas the right part corresponds to the experiment with the sample cut in the direction transverse to the grain. The numbers on the left of the high-speed registration images indicate the number of loading cycles (running number of impulse in the loading measuring bar) which one can see in Figure 7. It can be seen in Figure 10 that the samples cutting off parallel to the grain remain intact throughout the experiment, which is confirmed by the final state of such samples. Samples obtained by cutting in the direction perpendicular to the grain retain their integrity during the first loading cycle, however, cracks and gaps between the layers of wood appear in the second cycle, which grow and progress in subsequent loading cycles, leading to the separation of the sample into parts.

It can be noted that for the same load amplitude, the samples obtained by cutting in the direction of the fiber remain intact, while the samples cut perpendicular to the fiber exhibit failure already in the first loading cycle. 

**Figure 11:**
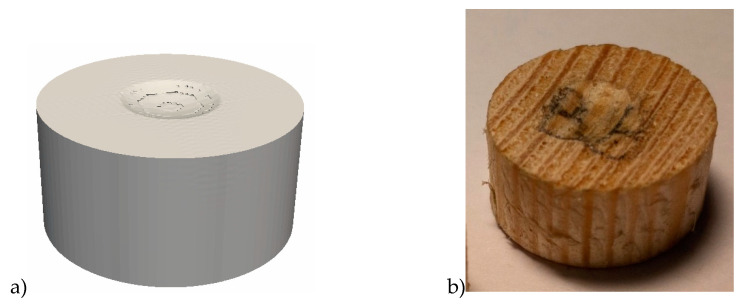
The shape of the imprints obtained by numerical modeling (**a**) and in the natural test (**b**).

A comparison of the shape of the imprint on the pine sample after the experiment on the dynamic indentation of a hemispherical indenter is shown in Figure 11.

**Figure 12:**
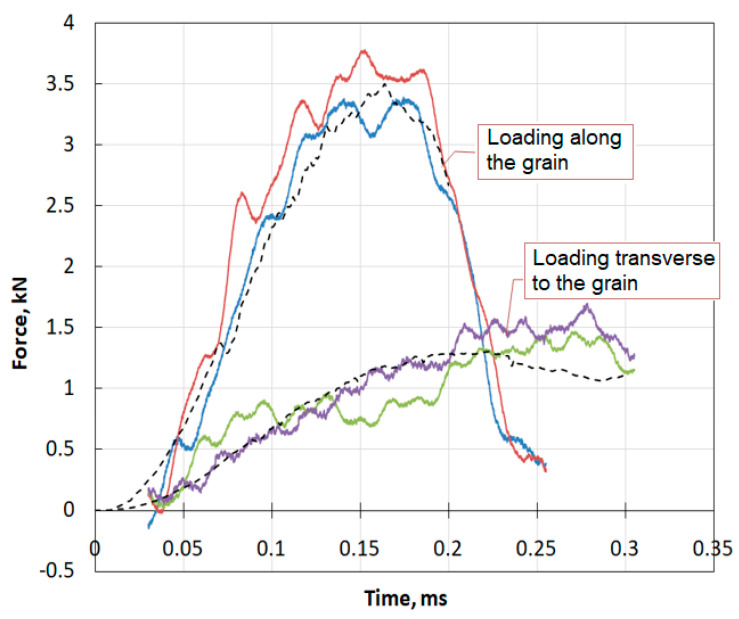
Comparison of experimental and calculated pulses in the supporting bar.

Figure 12 shows a comparison of the time dependences of the indentation resistance of pine sample recorded in the experiments (solid colored lines) with the data obtained by numerical modeling (black dashed lines) during dynamic indentation of hemispherical indenter into specimen both along and across the grain.

It can be seen that the model used allows us to accurately reproduce the features of the deformation of real material, namely, the difference in its deformation behavior in different directions with respect to grain orientation.

The results obtained are in qualitative agreement with the data from previous studies of high strain rate behavior of wood [8,17]. Correction of some parameters of the MAT_WOOD model made it possible to describe the deformation behavior of the studied wood species with sufficient accuracy. A further increase in the quality of the mathematical model is possible by taking into account the effect of the strain rate in a wide range of its change, as well as taking into account the fracture of the material. In addition, it is necessary to conduct more complex model experiments to verify the fracture criteria, for example, high-speed penetration and perforation of wood plates.

## 5. Conclusions

Dynamic tests of lime-tree and pine were conducted. There is a strong anisotropy of the properties of the tested materials: the specimens exhibit the greatest strength under load applied along the grain, while the lowest strength is observed under loading transverse to the grain. The load branch module is non-linear and, as a rule, smaller than the unloading branch module (while maintaining the specimen integrity). At the specimen cutting angle of 90° with respect to the direction of the fibers, the stress–strain diagram after reaching a certain threshold stress value (about 8 MPa for lime-tree and about 10 MPa for pine) is close to the ideal plastic diagram. At 0° cutting angle, the initial section of the diagrams is close to linear. That is, an elastic deformation occurs. However, after reaching an ultimate stress value (yield strength), the diagram becomes nonlinear with stress relaxation. Especially such behavior is observed in the experiments in which the failure of specimens occurs.

Using a modification of the SHPB method, experiments were performed on the dynamic indentation of indenters with a hemispherical head into the samples parallel and perpendicular to the direction of the fibers. Dependences of resistance to penetration on time are constructed. In the direction of the fiber (along the grain), the resistance force was more than three-times greater than in the transverse direction. The results of the natural experiment were compared with the results of numerical modeling, in which the wood behavior was described by the MAT_WOOD model from the LS-DYNA software, in which some of the parameters were obtained experimentally. A good coincidence was observed.

## Figures and Tables

**Table 1:** The physical parameters of tested materials.

Wood Species	Lime-Tree		Pine	
Parameter	Along the Grain	Transverse to the Grain	Along the Grain	Transverse to the Grain
Density, g/cm^3^	0.505 ± 0.02	0.512 ± 0.015	0.435 ± 0.01	0.484 ± 0.015
Moisture content, %	7.5 ± 0.2	7.9 ± 0.3	6.8 ± 0.3	7.1 ± 0.2

**Table 2:** Main mechanical properties of pine for model identification.

Designation	Value
Density	450 kg/m^3^
Modulus of elasticity in the direction of the grain	10,500 MPa
Flow stress during compression in the direction of the grain	80 MPa
The modulus of elasticity in the direction perpendicular to the grain	246 MPa
Flow stress during compression in the direction perpendicular to the grain	10 MPa
